# Effect of CYP2D6 pharmacogenetic phenotype and phenoconversion on serum concentrations of antidepressants and antipsychotics: a retrospective cohort study

**DOI:** 10.1007/s11096-023-01588-8

**Published:** 2023-05-11

**Authors:** Maike Scherf-Clavel, Amelie Frantz, Andreas Eckert, Heike Weber, Stefan Unterecker, Jürgen Deckert, Andreas Reif, Martina Hahn

**Affiliations:** 1 Department of Psychiatry, Psychosomatics and Psychotherapy, Center of Mental Health, University Hospital of Würzburg, Margarete-Höppel-Platz 1, 97080 Würzburg, Germany; 2 Department of Psychiatry, Psychosomatic Medicine and Psychotherapy, University Hospital Frankfurt, 60528 Frankfurt, Germany; 3 Department of Mental Health, Varisano Hospital Frankfurt Hoechst, Frankfurt, Germany

**Keywords:** Antidepressive agents, Antipsychotic agents, Drug interactions, Pharmacogenetics, Pharmacokinetics, Phenoconversion

## Abstract

**Background:**

Pharmacogenetics (PGx), especially in regard to *CYP2D6*, is gaining more importance in routine clinical settings. Including phenoconversion effects (PC) in result interpretation could maximize its potential benefits. However, studies on genetics of pharmacokinetic genes including the functional enzyme status are lacking.

**Aim:**

The retrospective analyses of clinical routine data aimed to investigating how the CYP2D6 functional enzyme status affects serum concentrations and metabolite-to-parent ratios of seven common psychotropic drugs and allows an evaluation of the relevance of this information for patient care.

**Method:**

Two patient cohorts (total n = 316; 44.2 ± 15.4 years) were investigated for the CYP2D6 functional enzyme status and its associations with drug exposure and metabolism of venlafaxine, amitriptyline, mirtazapine, sertraline, escitalopram, risperidone and quetiapine.

**Results:**

We found an increase in intermediate and poor metabolizers, as well as a decrease in normal metabolizers of CYP2D6 when including PC. Moreover, we found associations between amitriptyline exposure with the phenoconversion-corrected activity score of CYP2D6 (Spearman correlation; *p* = 0.03), and risperidone exposure with CYP2D6 functional enzyme status (Kruskal–Wallis test; *p* = 0.01), as well as between metabolite-to-parent ratio of venlafaxine and risperidone with CYP2D6 functional enzyme status (Kruskal–Wallis test; *p* < 0.001; *p* = 0.05).

**Conclusion:**

The data stress the relevance of PC-informed PGx in psychopharmacological treatment and suggest that PC should be included in PGx result interpretation when PGx is implemented in routine clinical care, especially before initiating amitriptyline- or risperidone-treatment, to start with a dose adequate to the respective CYP2D6 functional enzyme status. Moreover, PGx and therapeutic drug monitoring should be used complementary but not alternatively.

**Supplementary Information:**

The online version contains supplementary material available at 10.1007/s11096-023-01588-8.

## Impact statements

- Phenoconversion effects are currently underestimated in clinical practice, but should be included in PGx result interpretation in routine clinical care.
- To maximize the potential benefits of PGx testing, specific expertise in PGx is required, for example by embedding clinical pharmacists in clinical routine.
- For amitriptyline and risperidone treatment PC-informed PGx before initiating medication is recommended, to start with a dose adequate to the respective CYP2D6 functional enzyme status.

## Introduction

Pharmacogenetics (PGx) investigates how genetic polymorphisms affect treatment response or adverse effects and is greatly contributing to the advent of *precision medicine* approaches [1–3]. Cytochrome P450 (CYP) enzymes show a high variability in their activity due to genetic variations. Therefore, PGx assessment, especially in relation to *CYP2D6* and *CYP2C19*, has an increasingly recognized, and critical role in determining psychopharmacological treatment [4–6]. Accordingly, clinical recommendations for patients treated with tricyclic antidepressants (TCA) and selective serotonin reuptake inhibitors (SSRI), specify how to adjust dosages according to the patient’s *CYP2D6* and *CYP2C19* phenotypes [7–10]. Given that TCA and SSRI, but also other psychotropic drugs, are mainly metabolized by CYP2D6 and/or CYP2C19 [11, 12], it can be hypothesized that clinical recommendations should be extended to other psychopharmacological compounds. However, studies investigating the effect of *CYP2D6* and *CYP2C19* phenotypes on serum concentrations and metabolism of psychopharmacological drugs in a clinical setting are lacking. In consequence, implementation of PGx in routine clinical care is in its infancy and research on how to optimize the use and implementation of PGx is essential [4].

For application in PGx routine clinical work, the diplotype and the corresponding phenotype of CYP2D6 and CYP2C19 are used [9, 10], even if the phenotype-definition is a dynamic assignment [13]. In addition, the phenotype is affected by concomitant drugs, as CYP2D6 and CYP2C19 are susceptible to enzyme inhibition and/or induction [14, 15]. This can lead to a discordance between the genotype-inferred phenotype and the clinically observed phenotype, which is termed a phenoconversion effect (PC) [16]. Therefore, a calculator tool was established to integrate standardized assessments of PC for CYP2D6 phenotypes in clinical practice [14, 16]. Depending on the inhibitory properties of the comedication, the activity score of CYP2D6 is multiplied by a corresponding factor leading to an adjusted activity score [14]. With respect to *CYP2C19*, there is no consensus on how to adjust phenotypes [14, 16, 17]. In a recent study on PC, CYP2D6 poor metabolizers (PM) had the largest increase in PC-corrected phenotypes [18], making the phenotype “poor metabolizer” much more common than expected from population data. In summary, PC are highly prevalent, but are not yet integrated into routine clinical processes [14]. Also, studies, reporting pharmacokinetics of the drugs with respect to the CYP2D6 functional enzyme status are mainly missing. One study on clozapine showed the relevance of nongenetic factors on serum concentrations [19]. In addition, a study on antipsychotics taken into account PC, showed that CYP2D6 functional status affected serum concentration of aripiprazole, haloperidol, risperidone, and zuclopenthixol; however, only a small number of patients were included in the analyses (n = 18/11/20/6) [20]. Regarding antidepressants, only one study is currently available, investigating nortriptyline and venlafaxine, but genotyping was limited to CYP2D6*3 and *4 [21].

### Aim

To address these prevailing PGx issues, the present study investigates how the CYP2D6 functional enzyme status affects serum concentrations and metabolite-to-parent ratios of seven psychotropic drugs and allows an evaluation of the relevance of this information for patient care.

### Ethics approval

The retrospective analysis of routine clinical data without additional explicit written informed consent was performed in accordance with a vote by the Wuerzburg Ethics committee (20220120 02) and the principles of the Declaration of Helsinki. The “Acceptance, Use, and Feasibility of Pharmacogenetic Testing in Psychiatry” (FACT-PGx) study was approved by the local ethics committee of the University of Frankfurt (2021-138) and carried out in accordance with the ethical principles of the Declaration of Helsinki version 2013. Written informed consent was obtained from each participant.

## Method

### Patients

#### Wuerzburg sample

Inpatients at the Department of Psychiatry, Psychosomatics and Psychotherapy of the University Hospital of Wuerzburg with available genotype data, as well as therapeutic drug monitoring (TDM) results were included in the analyses. Only patients older than 18 years were included. Genotyping of *CYP2D6* and *CYP2C19*, as well as TDM was performed as part of routine clinical care. Genotyping for *CYP2D6* was performed essentially according to recommendations of the German Genetic Diagnostics Commission [22, 23] and according to the procedures of the German Genetic Diagnostics Act with written informed consent. In the Wuerzburg sample 212 patients were included. Genotypes and serum concentrations were determined between January 2020 and December 2021.

#### Frankfurt sample

Inpatients admitted to the Department of Psychiatry, Psychosomatic Medicine and Psychotherapy of the University Hospital Frankfurt due to a depressive episode older than 18 years were genotyped for *CYP2D6* as part of the FACT-PGx study. TDM was performed as part of the clinical routine. Data of patients that took part in the FACT-PGx study with available TDM data were included in the analyses. In the Frankfurt sample 104 patients were included. Genotypes and serum concentrations were determined between July 2021 and March 2022.

### Genotyping and therapeutic drug monitoring

Genotyping of *CYP2D6* and TDM was performed at the Department of Psychiatry, Psychosomatics and Psychotherapy of the University Hospital of Würzburg.

Details about the methods can be find in Online Resource 1.

Phenotypes of CYP2D6 were determined according to the Clinical Pharmacogenetics Implementation Consortium (CPIC) specifications [13].

Dose-corrected serum concentrations (serum concentration/dose, CD) of the active moiety of the drug (serum concentration parent drug + active metabolite; CD_AM_), or the parent drug, depending on the relevance for treatment response [24], and metabolite-to-parent ratios (MPR) were calculated [24]. To avoid bias in case of multiple serum concentration determinations for one drug in the same patient, only the last determination per analyte was included in the analyses.

Dimensional outliers (≥ 3 SD from mean) from CD and MPR were set as missing data.

### Phenoconversion effects

Phenoconversion effects were assessed according to Cicali et al. [14]. The activity score of CYP2D6 in patients receiving a moderate and strong CYP2D6 inhibitor was multiplied with 0.5, and 0, respectively, and the corresponding adjusted phenotype (functional enzyme status; phenotype_PC_) was determined to the adjusted activity factor according to CPIC specifications [13, 14]. Concomitant drugs with the propensity to cause PC due to inhibitory or inducing effects on CYP2D6 were derived from the Flockhart table (Online Resource 2) [15]. As melperone and perazine potentially show CYP2D6 inhibitory effects, but were not listed in the Flockhart table, patients receiving melperone or perazine (n = 9) were excluded from the analyses [15, 25].

### Statistical analyses

Statistical analyses were conducted in R v4.0.4 [26].

To investigate if the CYP2D6 phenotypes differ from the functional enzyme status McNemar tests with continuity correction was performed; *p* < 0.05 was considered significant.

To investigate differences in CD and MPR depending on the CYP2D6 functional enzyme status Kruskal–Wallis tests were performed. Chi-square tests (expected sample size > 5) or Fisher's exact tests (expected sample size < 5) were performed to investigate the association between the functional enzyme status and serum concentrations below, above or within the therapeutic reference range [24] for the respective drug. Groups (below, above or within the therapeutic reference range) with less than five patients were excluded from analyses. One-tailored spearman correlation was performed to investigate the association between the PC-corrected activity score for CYP2D6 and CD of the drugs.

Benjamini–Hochberg correction was performed with a significance threshold of *p* < 0.05 in each analysis, as Bonferroni correction tends to be too conservative for genomic analysis as the data were not completely independent due to the linkage equilibrium [27].

## Results

### Patient samples

The combined sample comprised 316 patients, 212 from Wuerzburg, and 104 from Frankfurt. Patients were 44.2 ± 15.4 (mean ± standard deviation (SD)) years old, and 54.1% female. 144 patients were nonsmokers, 99 were smokers, and from 73 patients no information on smoker status was available. A more detailed demographic overview is given in Table 1.

**Table 1:** Demographic data of the patients included in the sample. Genotypic phenotypes were the phenotypes according to the PGx results. One phenotype per patient is given. The number of patients exceed the number of genotypic phenotypes as some genotypes were not clearly assignable to one phenotype. Results on phenoconversion effects (PC) are given per TDM request, as concomitant drugs with each TDM request affect the phenotype; thus, the number of phenotypes exceed the number of patients

	Combined sample		Wuerzburg sample		Frankfurt sample	
	N	Mean ± SD (range)	N	Mean ± SD (range)	N	Mean ± SD (range)
Included patients	316		212		104	
Age [years]	316	44.2 ± 15.4 (18–84)	212	44.9 ± 15.5 (18–84)	104	42.7 ± 15.4 (19–77)
Male/Female	145/171		93/119		52/52	
Nonsmoker/Smoker	144/99		89/54		55/45	
Nonsmoker M/F	57/87		32/57		25/30	
Smoker M/F	56/43		29/25		27/18	
*Genotypic phenotypes*						
CYP2D6	291		194		97	
UM/RM/NM/IM/PM (%)	7/0/155/113/16 (2.4/0/53.3/38.8/5.5)		2/0/104/78/10 (1.0/0/53.6/40.2/5.2)		5/0/51/35/6 (5.2/0/52.6/36.1/6.2)	
*Phenotypes per TDM request*						
CYP2D6 (nonPC/PC)	588/540		412/383		176/157	
nonPC: UM/RM/NM/IM/PM	13/0/324/222/29		4/0/230/160/18		9/0/94/62/11	
PC: UM_PC_/RM_PC_/NM_PC_/IM_PC_/PM_PC_	5/0/242/225/68		3/0/191/146/43		2/0/51/79/25	

*N*, number of patients; *(%)*, percentage number; *SD*, standard deviation; *M*, male; *F*, female; *nonPC*, non-phenoconversion; *PC*, phenoconversion; *UM*, ultrarapid metabolizer; *RM*, rapid metabolizer; *NM*, normal metabolizer; *IM*, intermediate metabolizer; *PM*, poor metabolizer

Administered drugs with serum concentration determinations are listed in Online Resource 3. Patients received between 0 and 18 additional drugs in combination (mean ± SD 4.1 ± 3.5). For power reasons, only patients who received venlafaxine (N = 117), amitriptyline (N = 100), mirtazapine (N = 85), sertraline (N = 64), escitalopram (N = 52), risperidone (N = 73), and quetiapine (N = 125) were included. Demographic data of these patients are given in Table 2.

**Table 2:** Demographic data of the patients included in the samples for each drug, and results of the respective analyses. Significant results are shown in bold

	Venlafaxine		Amitriptyline		Mirtazapine		Sertraline		Escitalopram		Risperidone		Quetiapine	
	N	Mean ± SD (range)	N	Mean ± SD (range)	N	Mean ± SD (range)	N	Mean ± SD (range)	N	Mean ± SD (range)	N	Mean ± SD (range)	N	Mean ± SD (range)
Included patients	117		100		85		64		52		73		125	
Age [years]	117	46.8 ± 14.9 (18–74)	100	47.7 ± 14.2 (20–82)	85	51.0 ± 16.1 (18–84)	64	39.4 ± 16.1 (18–84)	52	42.0 ± 4.0 (19–80)	73	48.7 ± 14.2 (20–75)	125	46.8 ± 15.8 (19–82)
Male/Female	57/60		49/51		44/41		21/43		20/32		34/39		61/64	
Nonsmoker/Smoker	45/49		45/31		40/18		35/18		27/13		33/19		46/38	

	Venlafaxine		Amitriptyline		Mirtazapine		Sertraline		Escitalopram		Risperidone		Quetiapine	
	N	*p*-value	N	*p*-value	N	*p*-value	N	*p*-value	N	*p*-value	N	*p*-value	N	*p*-value
*CYP2D6*														
UM_PC_/RM_PC_/NM_PC_/IM_PC_/PM_PC_	2/0/56/32/9		0/0/42/29/9		1/0/43/27/5		0/0/21/31/5		0/0/22/15/7		0/0/34/23/3		2/0/50/43/11	
CD		0.07		0.10		0.37		0.39		0.87		**0.01**		0.08
MPR		3.5*10^**–9**^		0.37		0.52				0.13		*0.05*		0.23
Reference range		0.85		0.18		0.72				0.63		0.21		0.44
Activity score		0.06		**0.03**		0.08		0.43		0.48		**0.008**		0.43

*N*, number of patients; *SD*, standard deviation; *UM*, ultrarapid metabolizer; *PC*, phenoconversion; *RM*, rapid metabolizer; *NM*, normal metabolizer; *IM*, intermediate metabolizer; *PM*, poor metabolizer; *CD*, dose-corrected serum concentration; *MPR*, metabolite-to-parent ratio

### Phenoconversion effect

**Fig. 1:**
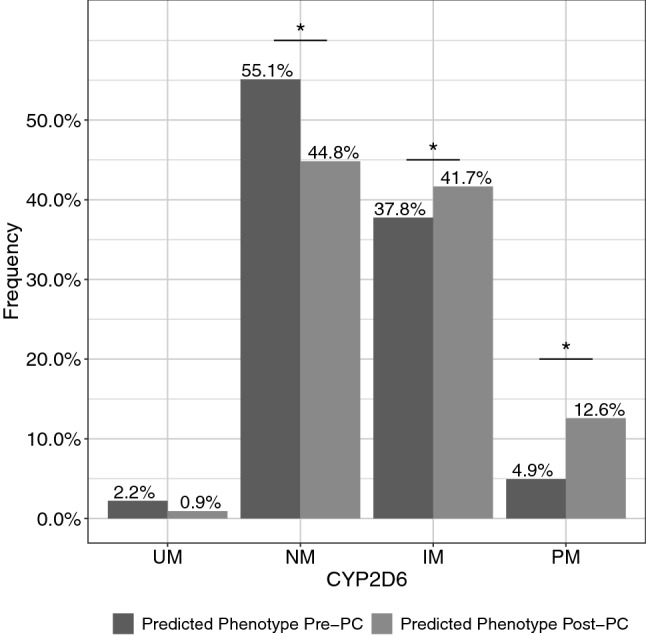
Frequencies of predicted CYP2D6 phenotype before (pre) and after (post) including phenoconversion effects. (UM, ultrarapid metabolizer; NM, normal metabolizer; IM, intermediate metabolizer; PM, poor metabolizer)

While at baseline, 55.1% of the patients were classified as CYP2D6 normal metabolizers (NM), after accounting for PC only 44.8% were still classified as NM_PC_ (*p* < 0.001). Moreover, the number of intermediate metabolizers (IM) significantly increased from 37.8 to 41.7% (*p* < 0.001), and also the number of poor metabolizers (PM) significantly increased from 4.9 to 12.6% (*p* < 0.001). On the contrary, the number of ultrarapid metabolizers (UM) decreased from 2.2 to 0.9%, but this decrease was not significant (*p* = 0.08) (Fig. 1).

### Venlafaxine

**Fig. 2:**
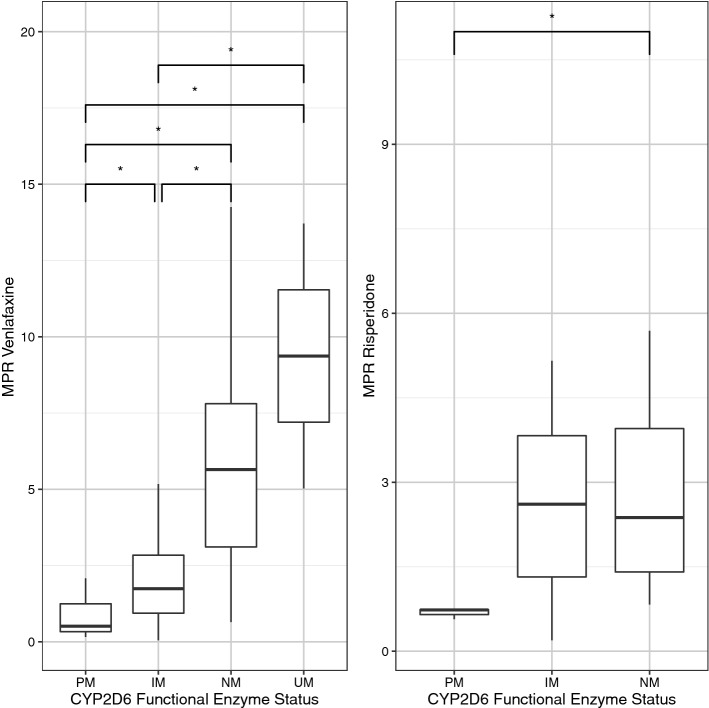
MPR of venlafaxine, as well as of risperidone were associated with CYP2D6. (UM, ultrarapid metabolizer; NM, normal metabolizer; IM, intermediate metabolizer; PM, poor metabolizer)

CD_AM_ of venlafaxine was not associated with the CYP2D6 functional enzyme status (*p* = 0.07), but MPR was associated (*p* = 3.5*10^–9^) with higher levels in UM_PC_ compared to IM_PC_ (*p* = 0.024) and PM_PC_ (*p* = 0.036), in NM_PC_ compared to IM_PC_ (*p* = 1.6*10^–6^) and PM_PC_ (*p* = 1.5*10^–5^), and in IM_PC_ compared to PM_PC_ (*p* = 0.011) (Fig. 2). The CYP2D6 functional enzyme status was not associated with serum concentrations below, above or within the therapeutic reference range (*p* = 0.85), nor was the PC-corrected activity score of CYP2D6 associated with CD_AM_ (*p* = 0.06).

### Amitriptyline

**Fig. 3:**
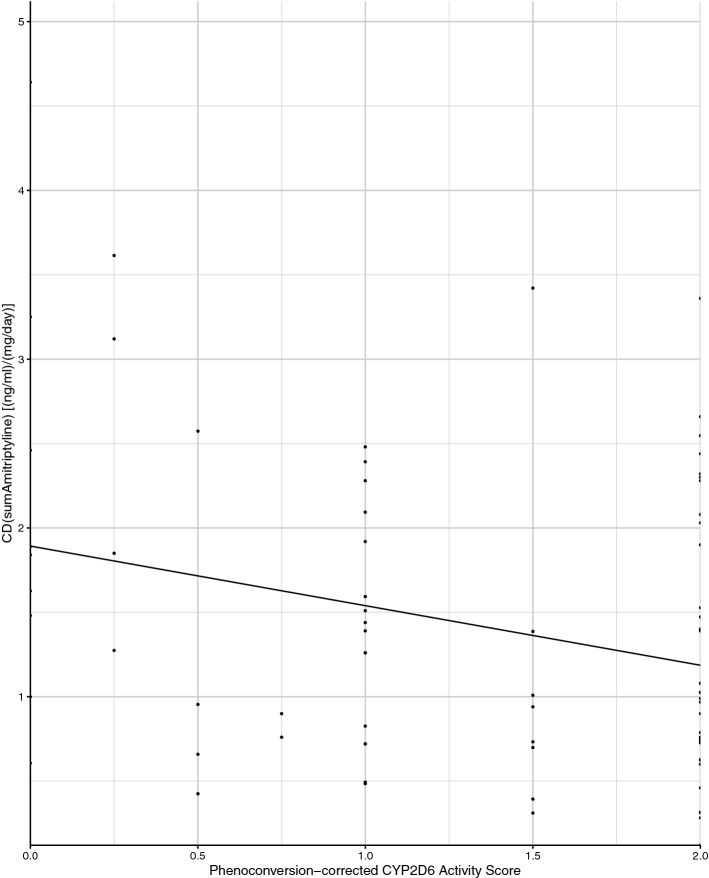
CD of amitriptyline was associated with the phenoconversion-corrected activity score of CYP2D6

CD_AM_ and MPR of amitriptyline were not associated with the CYP2D6 functional enzyme status (*p* = 0.10, *p* = 0.37); moreover, the CYP2D6 functional enzyme status was not associated with serum concentrations below, above or within the therapeutic reference range (*p* = 0.18). However, the PC-corrected activity score of CYP2D6 was associated with CD_AM_ (*p* = 0.03) (Fig. 3).

### Mirtazapine

CD, as well as MPR of mirtazapine were not associated with the CYP2D6 functional enzyme status (*p* = 0.37, *p* = 0.52). Serum concentrations of mirtazapine within, above and below the respective therapeutic reference range were not associated with the CYP2D6 functional enzyme status (*p* = 0.72), nor was the PC-corrected activity score of CYP2D6 associated with CD (*p* = 0.08).

### Sertraline

CD of sertraline was not associated with the CYP2D6 functional enzyme status (*p* = 0.39), and the PC-corrected activity score of CYP2D6 was not associated with CD (*p* = 0.43).

No metabolite was recorded, thus, analyses on MPR were not possible. In addition, only one and no patient showed serum concentrations below and above the therapeutic reference range, respectively; therefore further analyses could not be conducted.

### Escitalopram

CD and MPR of escitalopram were not associated with the CYP2D6 functional enzyme status (*p* = 0.87, *p* = 0.13). Serum concentrations of escitalopram within, above and below the respective therapeutic reference range were not associated with the CYP2D6 functional enzyme status (*p* = 0.63), nor was the PC-corrected activity score of CYP2D6 associated with CD (*p* = 0.48).

### Risperidone

**Fig. 4:**
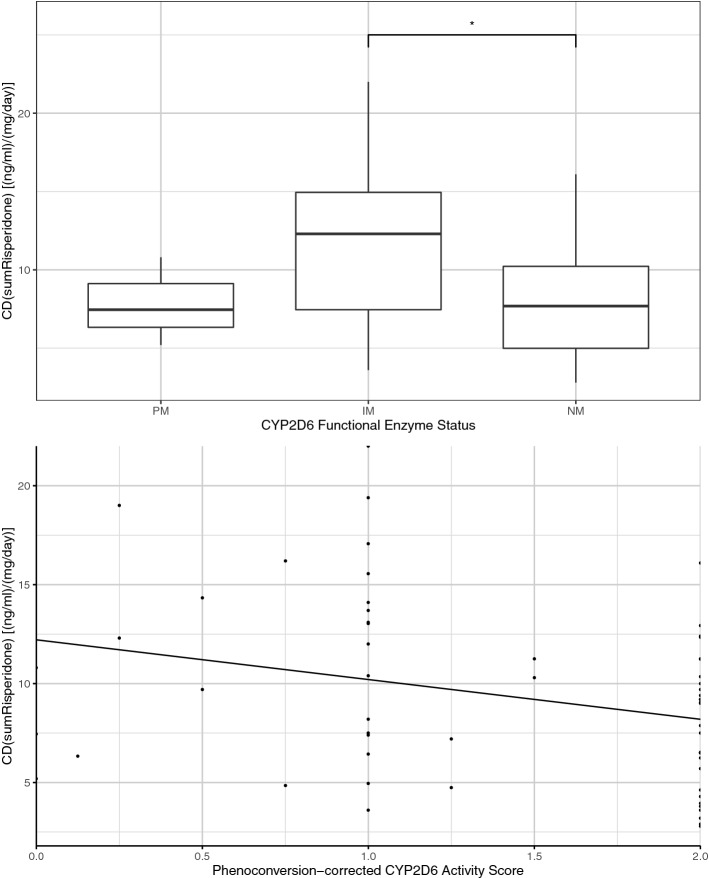
CD of risperidone was associated with the functional metabolizer status of CYP2D6, as well as the phenoconversion-corrected activity score of CYP2D6. (NM, normal metabolizer; IM, intermediate metabolizer; PM, poor metabolizer)

CD_AM_ of risperidone was associated with the CYP2D6 functional enzyme status (*p* = 0.01); pairwise comparison showed that CD_AM_ was higher in IM_PC_ compared to NM_PC_ (*p* = 0.01) (Fig. 4). MPR also was associated with the CYP2D6 functional enzyme status (*p* = 0.05) with significant higher levels in NM_PC_ compared to PM_PC_ (*p* = 0.02); moreover, unadjusted pairwise comparison showed higher MPR in IM_PC_ compared to PM_PC_ (*p* = 0.05) (Fig. 2). The CYP2D6 functional enzyme status was not associated with serum concentrations below, above or within the therapeutic reference range (*p* = 0.21). However, the PC-corrected activity score of CYP2D6 was associated with CD_AM_ (*p* = 0.008) (Fig. 4).

### Quetiapine

CD and MPR of quetiapine were not associated with the CYP2D6 functional enzyme status (*p* = 0.08, *p* = 0.23). Also, the CYP2D6 functional enzyme status was not associated with the therapeutic reference range (*p* = 0.44, *p* = 0.73). Moreover, the PC-corrected activity score of CYP2D6 was not associated with CD (*p* = 0.43).

## Discussion

In this naturalistic study, the association between CYP2D6 functional enzyme status and the pharmacokinetics of seven antidepressant and antipsychotic drugs in a routine clinical setting was investigated. The findings reveal a significant increase in CYP2D6 IM and PM, and, in contrast, a significant decrease in CYP2D6 NM when accounting for PC. Moreover, significant associations between drug exposure of amitriptyline and risperidone with CYP2D6 functional metabolizer status were found. In addition, the metabolism of venlafaxine and risperidone was associated with the CYP2D6 functional metabolizer status. For escitalopram, sertraline, quetiapine and mirtazapine, drug interactions with CYP2D6 inhibitors may not be clinically relevant.

The results on PC were in concordance with previous studies, reporting an increase in IM and PM [17, 18]. Thus, without accounting for PC, the number of PM and IM was underestimated [28]. For the first time it was reported that the number of CYP2D6 NM was overestimated when PC was not considered. In consequence, without considering PC, there is a risk of potentially inaccurate dose adjustments. Thus, even if some complexity is added when including PC for CYP2D6 in PGx result interpretation, this may improve the prediction of pharmacokinetics, as the more specific functional status of the enzyme is taken into account. However, prerequisite for including PC is having an accurate medication list [28]. Thus, to maximize the potential benefits of PGx testing, specific expertise in PGx is required, for example by embedding clinical pharmacists in clinical routine [18, 29].

For the first time, association analyses were conducted, including the CYP2D6 functional enzyme status. Venlafaxine is mainly metabolized by CYP2D6 to its active metabolite *O*-desmethylvenlafaxine, while CYP2C19, together with CYP3A4, is mainly responsible for the formation of *N*-desmethylvenlafaxine [30, 31]. In the present study, in accordance with previous studies, an influence of *CYP2D6* on the conversion from venlafaxine to *O*-desmethylvenlafaxine, but not on the active moiety serum concentration was reported [32–36]. One study also reported higher *N*-desmethylvenlafaxine concentrations in CYP2D6 PM compared to NM patients [33], pointing to an additional pathway in PM patients beside CYP2D6 that is increased as a consequence of the CYP2D6 phenotype, which may result in adverse drug effects [33, 37].

Focusing on amitriptyline, and in accordance with clinical guidelines [7, 8, 10, 38, 39], the results confirm an influence of *CYP2D6* on its metabolism. In contrast to a previous study, reporting an association with CYP2D6 phenotypes [32], CD_AM_ was not associated with CYP2D6 functional enzyme status, but rather with the PC-corrected activity score of CYP2D6. Without including PC on *CYP2D6*, CD_AM_ of amitriptyline was associated with *CYP2D6* phenotypes. We, therefore, support previous recommendations to reduce initial doses in PM_PC_ patients, and to use TDM to appropriately adjust doses of amitriptyline. However, there are only limited studies available on PGx in amitriptyline-treated patients that assess drug exposure, given that most studies to date have focused on treatment response [40].

Beside antidepressants, two antipsychotics that are recommended for antidepressant augmentation were investigated. For risperidone it was found that CD_AM_ was associated with *CYP2D6*, with higher CD_AM_ in IM_PC_ compared to NM_PC_ patients, and that the PC-corrected activity score of CYP2D6 was associated with CD_AM_. Moreover, MPR was associated with *CYP2D6* with significant higher levels in NM_PC_ compared to PM_PC_. Therefore, *CYP2D6* affect not only the active moiety serum concentration, but also the metabolism of risperidone. For risperidone-treated patients, current clinical recommendations are given for *CYP2D6* by the Dutch Pharmacogenetics Working Group (DPWG) [38]. Nevertheless, no recommendation is given for IM patients [41]. In IM_PC_, as well as in PM_PC_ patients, we suggest using TDM to titrate dose to prevent serum concentration above the therapeutic reference range.

As CYP2D6 is not mainly involved in the metabolism of mirtazapine, sertraline, escitalopram and quetiapine, the functional enzyme status was not associated with either the CD nor with MPR of these drugs [24]. Moreover, drug-drug interactions with respect to CYP2D6 may be negligible for these drugs.

In consequence, PGx is not an alternative to TDM, but both tools should be used complementary for providing *precision medicine* [42]. PGx information can be used predictive in lifetime; in contrast, TDM also captures the influence of environmental factors, for example smoking or comedications [42]. Including PC in PGx result interpretation improves the benefit of PGx testing, even if it adds some complexity. However, genotypes are static information, but the functional enzyme status is a dynamic information that should be continually evaluated throughout the patient´s lifetime, as it depends on concomitant medications [28].

### Strengths and limitations

The present retrospective study in two independent cohorts provides real life data from a naturalistic setting and in consequence, the results are relevant to clinical settings. Since the phenotypes are proposed methods for prescribing drugs, analyses were performed on phenotypes, even if such phenotype definitions may change over time. Most important, PC for CYP2D6 were included, and consequently, association analyses were done with the functional enzyme status to maximize the potential benefits of PGx testing. However, there were limitations in the analyses. *CYP2C19* was not included as, no standardization on how to adjust the phenotype based on genotype and inhibitor use is available [14, 16]. There were only a limited number of patients included in each of the analyses, however, such real-life data on PGx are limited; therefore, the results are more important for supporting routine PGx-testing to provide *precision medicine*. The analyses were not controlled for age, sex and smoking status. Clinical data were not available in either of the cohorts and adverse drug effects were not recorded; however, as dosing was adjusted to serum concentrations and PGx results, serum concentrations outside the therapeutic reference range were rare and severe adverse drug effects were not expected [32]. Moreover, inclusion criteria in both samples were not the same; the Wuerzburg cohort included all patients, regardless of their diagnosis, from which TDM was available and PGx was requested; in contrast, in the Frankfurt cohort only patients suffering from a depressive episode were included. The cohorts were not limited to Caucasians, but patients with different ethnicities were included, but ethnicity was not recorded. Nevertheless, due to the pharmacokinetic aspect of the aim of this analysis, these limitations did not affect the results.

## Conclusion

Whilst PGx testing, in addition to TDM, offers the next useful step to provide *precision medicine,* including PC in result interpretation could improve the prediction of the therapy results, even if complexity is added. Not taking into account PC underestimates the number of CYP2D6 PM and IM, and overestimates the number of NM. CYP2D6 functional enzyme status was associated with the metabolism of venlafaxine, but not with the active moiety, pointing to an association with adverse drug effects. The results also support previous recommendations to reduce starting doses of amitriptyline in CYP2D6 PM_PC_, and to use TDM to adjust doses. *CYP2D6* affected not only the active moiety serum concentration of risperidone, but also its metabolism. Therefore, we recommend to perform PGx before initiating amitriptyline or risperidone. The findings stress the relevance that in *precision medicine* in psychiatry, PGx and TDM should be used in a complementary manner (PGx informed TDM).

### Supplementary Information

Below is the link to the electronic supplementary material.

Supplementary file1 (PDF 674 KB)

Supplementary file2 (PDF 399 KB)

Supplementary file3 (PDF 414 KB)
