# Supplementary material for: Effect of CYP2D6 pharmacogenetic phenotype and phenoconversion on serum concentrations of antidepressants and antipsychotics: a retrospective cohort study
Source: Int J Clin Pharm. 2023 May 11;45(5):1107–17. doi: 10.1007/s11096-023-01588-8 (PMC10600053; doi:10.1007/s11096-023-01588-8)
Supplement: Supplementary file 1 — Supplementary file1 (PDF 674 KB) [file 11096_2023_1588_MOESM1_ESM.pdf]

International Journal of Clinical Pharmacy

Online Resource 1

to

Effect of CYP2D6 pharmacogenetic phenotype and phenoconversion on serum concentrations of antidepressants and antipsychotics - a retrospective cohort study

Maike Scherf-Clavel, PhD[1], Amelie Frantz[2], Andreas Eckert, MD[2], Heike Weber, PhD[1,2], Stefan Unterecker, MD[1], Jürgen Deckert, MD[1], Andreas Reif, MD[2], Martina Hahn, PhD[2,3]

[1] Department of Psychiatry, Psychosomatics and Psychotherapy, Center of Mental Health, University Hospital of Würzburg, 97080 Würzburg, Germany
[2] Department of Psychiatry, Psychosomatic Medicine and Psychotherapy, University Hospital Frankfurt, 60528 Frankfurt, Germany
[3] Department of mental health, varisano Hospital Frankfurt Hoechst, Germany

Corresponding author:

Dr. rer. nat. Maike Scherf-Clavel
Department of Psychiatry, Psychosomatics and Psychotherapy
University Hospital of Würzburg
Margarete-Höppel-Platz 1
97080 Würzburg, Germany
Tel.: +49/931/201 77546
Fax: +49/931/201 77262
E-Mail: Scherf_M@ukw.de

Genotyping

Genotyping of relevant gene variants in *CYP2D6* (single nucleotide polymorphisms (SNP) and star allele coverage; Table 1) was performed on a MassArray Analyzer 4 system (Agena Bioscience GmbH, Hamburg, Germany) based on a self-designed panel using SpectroCHIP®-96 Arrays and the iPLEX® Pro chemistry following the instructions supplied by the manufacturer. Due to specificity issues, a restriction fragment length polymorphism method was performed to determine rs3892097, rs16947, and rs1080995. Primer sequences are available on request. Moreover, copy number variations (CNV) were determined using the CYP2D6 RealFast™ CNV Assay, provided by ViennaLab Diagnostics GmbH, Vienna, Austria [1]. The laboratory was certified by a quality control program [2].

Haplotypes were defined for all analysed SNPs according to gene specific haplotype tables from the PharmVar homepage (https://www.pharmvar.org/genes; Table 1). Phenotypes of CYP2D6 were determined according to the Clinical Pharmacogenetics Implementation Consortium (CPIC) specifications [3].

Table 1 Haplotype table giving the combination of the SNPs for the according haplotypes.

	HAPLOTYPE	rs1080985	rs28735595	rs1065852	rs1080995	rs28371706	rs5030655	rs3892097	rs35742686	rs5030656	rs16947	rs1135840
CYP2D6	*1	G	T	G	C	G	A	G	T	I	G	C
	*2A	C	C	G	G	G	A	G	T	I	A	G
	*2B	G	T	G	C	G	A	G	T	I	A	G
	*2C	C	C	G	G	G	A	G	T	I	G	G
	*3	G	T	G	C	G	A	G	D	I	G	C
	*4	G	T	A	C	G	A	A	T	I	G	G
	*4J	G	T	A	C	G	A	A	T	I	G	C
	*4K	G	T	A	C	G	A	A	T	I	A	G
	*4M	G	C	G	C	G	A	A	T	I	G	C
	*4N	G	C	A	C	G	A	A	T	I	G	G
	*4P	G	C	A	C	G	A	A	T	I	G	C
	*5					Complete Gene Deletion						
	*6A	G	T	G	C	G	D	G	T	I	G	C

*6C	G	T	G	C	G	D	G	T	I	G	G
*9	G	T	G	C	G	A	G	T	D	G	C
*10A	G	T	A	C	G	A	G	T	I	G	G
*10B	G	C	A	C	G	A	G	T	I	G	G
*10C	G	C	A	C	G	A	G	T	I	G	C
*14	G	T	G	G	G	A	G	T	I	A	G
*17	G	T	G	C	A	A	G	T	I	A	G
*34	G	T	G	C	G	A	G	T	I	A	C
*35A	C	T	G	C	G	A	G	T	I	A	G
*35B	C	C	G	C	G	A	G	T	I	A	G
*39	G	T	G	C	G	A	G	T	I	G	G
*41	G	C	G	G	G	A	G	T	I	A	G
*46A	G	C	G	C	G	A	G	T	I	A	G
*46B	G	C	G	C	G	A	G	T	I	G	G
*46C	G	C	G	C	G	A	G	T	I	G	C
*58	G	C	G	G	A	A	G	T	I	A	G
*64	G	C	A	C	A	A	G	T	I	G	G
*69	G	C	A	C	G	A	G	T	I	A	G
*71	C	T	G	C	G	A	G	T	I	G	C
*82	G	T	G	C	T	A	G	T	I	G	C
*88	G	T	G	G	G	A	G	T	I	G	G
*114	G	T	A	C	G	A	G	T	I	A	G

Therapeutic drug monitoring

Serum concentrations were determined according to the Arbeitsgemeinschaft für Neuropsychopharmakologie und Pharmakopsychiatrie (AGNP)-TDM expert group consensus guideline [4]. Blood was drawn at trough concentrations at steady-state [4]. Serum concentrations of the drugs and their metabolites were determined using liquid chromatography tandem mass spectrometry (LC-MS/MS) methods using MassTox® TDM Serie A,

provided by Chromsystems (Chromsystems Instruments & Chemicals GmbH, Gräfeling, Germany) [5]. The laboratory was certified by a quality control program [2]. Age, sex, smoking status, concomitant medication and the daily dose of the drugs were obtained from the treating physician.

References

1. ViennaLab Diagnostics GmbH. CYP2D6 RealFast CNV Assay. 2021
2. INSTAND Gesellschaft zur Förderung der Qualitätssicherung in medizinischen Laboratorien e. V. 2020. https://www.instand-ev.de/ueber-instand-ev/instand-ev.html; Accessed 10 Feb, 2020
3. CPIC - Clinical Pharmacogenetics Implementation Consortium. 2021. https://cpicpgx.org/; Accessed 08 March, 2022
4. Hiemke C, Bergemann N, Clement HW et al. Consensus guidelines for therapeutic drug monitoring in neuropsychopharmacology: update 2017. Pharmacopsychiatry 2018; 51: 9-62
5. Chromsystems Instrumental & Chemicals GmbH. Effizientes Drug Monitoring mit LC-MS/MS. www.Chromsystems.de. 2019
