# Supplementary material for: Effect of CYP2D6 pharmacogenetic phenotype and phenoconversion on serum concentrations of antidepressants and antipsychotics: a retrospective cohort study
Source: Int J Clin Pharm. 2023 May 11;45(5):1107–17. doi: 10.1007/s11096-023-01588-8 (PMC10600053; doi:10.1007/s11096-023-01588-8)
Supplement: Supplementary file 2 — Supplementary file2 (PDF 399 KB) [file 11096_2023_1588_MOESM2_ESM.pdf]

International Journal of Clinical Pharmacy

Online Resource 2

to

Effect of CYP2D6 pharmacogenetic phenotype and phenoconversion on serum concentrations of antidepressants and antipsychotics - a retrospective cohort study

Maike Scherf-Clavel, PhD[1], Amelie Frantz[2], Andreas Eckert, MD[2], Heike Weber, PhD[1,2], Stefan Unterecker, MD[1], Jürgen Deckert, MD[1], Andreas Reif, MD[2], Martina Hahn, PhD[2,3]

[1] Department of Psychiatry, Psychosomatics and Psychotherapy, Center of Mental Health, University Hospital of Würzburg, 97080 Würzburg, Germany
[2] Department of Psychiatry, Psychosomatic Medicine and Psychotherapy, University Hospital Frankfurt, 60528 Frankfurt, Germany
[3] Department of mental health, varisano Hospital Frankfurt Hoechst, Germany

Corresponding author:

Dr. rer. nat. Maike Scherf-Clavel
Department of Psychiatry, Psychosomatics and Psychotherapy
University Hospital of Würzburg
Margarete-Höppel-Platz 1
97080 Würzburg, Germany
Tel.: +49/931/201 77546
Fax: +49/931/201 77262
E-Mail: Scherf_M@ukw.de

Table 2 Drugs with the propensity to cause phenoconversion due to inhibitory or inducing effects on CYP2D6 according to the Flockhart table [The Flockhart Cytochrome P450 Drug-Drug Interaction Table. Updated 2021. https://drug-interactions.medicine.iu.edu/; cited: January, 12, 2022].

CYP2D6	
**Inhibitors**	**Inducers**
STRONG	Dexamethasone
Bupropion	Oritavancin
Cinacalcet	Rifampin
Fluoxetine	
Paroxetine	
Quinidine	
MODERATE	
Abiraterone	
Doxepin	
Duloxetine	
Halofantrine	
Lorcaserin	
Moclobemide	
Rolapitant	
Terbinafine	
WEAK	
Amiodarone	
Celecoxib	
Cimetidine	
Citalopram	
Clobazam	
Clomipramine	
Diphenhydramine	
Escitalopram	
Levomepromazine	
Sertraline	
