# Supplementary material for: Effect of CYP2D6 pharmacogenetic phenotype and phenoconversion on serum concentrations of antidepressants and antipsychotics: a retrospective cohort study
Source: Int J Clin Pharm. 2023 May 11;45(5):1107–17. doi: 10.1007/s11096-023-01588-8 (PMC10600053; doi:10.1007/s11096-023-01588-8)
Supplement: Supplementary file 3 — Supplementary file3 (PDF 414 KB) [file 11096_2023_1588_MOESM3_ESM.pdf]

International Journal of Clinical Pharmacy

Online Resource 3

to

Effect of CYP2D6 pharmacogenetic phenotype and phenoconversion on serum concentrations of antidepressants and antipsychotics - a retrospective cohort study

Maike Scherf-Clavel, PhD[1], Amelie Frantz[2], Andreas Eckert, MD[2], Heike Weber, PhD[1,2], Stefan Unterecker, MD[1], Jürgen Deckert, MD[1], Andreas Reif, MD[2], Martina Hahn, PhD[2,3]

[1] Department of Psychiatry, Psychosomatics and Psychotherapy, Center of Mental Health, University Hospital of Würzburg, 97080 Würzburg, Germany
[2] Department of Psychiatry, Psychosomatic Medicine and Psychotherapy, University Hospital Frankfurt, 60528 Frankfurt, Germany
[3] Department of mental health, varisano Hospital Frankfurt Hoechst, Germany

Corresponding author:

Dr. rer. nat. Maike Scherf-Clavel
Department of Psychiatry, Psychosomatics and Psychotherapy
University Hospital of Würzburg
Margarete-Höppel-Platz 1
97080 Würzburg, Germany
Tel.: +49/931/201 77546
Fax: +49/931/201 77262
E-Mail: Scherf_M@ukw.de

Table 3 Number (N) of administered psychiatric drugs with serum concentration determinations (TDM) in the combined sample.

ANTIDEPRESSANTS	N	ANTIPSYCHOTICS	N	ANTIEPILEPTICS	N
Venlafaxine	117	Quetiapine	125	Pregabaline	25
Amitriptyline	100	Risperidone	73	Pipamperone	20
Mirtazapine	85	Aripiprazole	32	Valproic Acid	15
Sertraline	64	Olanzapine	20	Lamotrigine	13
Escitalopram	52	Cariprazine	10	Oxcarbazepine	9
Bupropion	38	Clozapine	8	Gabapentine	3
Trazodon	32	Chlorprothixene	7	Carbamazepine	2
Duloxetine	28	Amisulpride	5	Topiramate	2
Clomipramine	27	Haloperidol	5	Levetiracetam	1
Milnacipran	19	Perazine	4		
Doxepine	16	Melperone	3		
Trimipramine	5	Benperidol	2		
Fluoxetine	4	Flupentixol	2		
Moclobemid	3	Fluphenazine	1		
Citalopram	2				
Maprotiline	1				
Opipramole	1				
